# How Sex Hormones Affect Migraine: An Interdisciplinary Preclinical Research Panel Review

**DOI:** 10.3390/jpm14020184

**Published:** 2024-02-07

**Authors:** Frederick Godley, John Meitzen, Hadas Nahman-Averbuch, Mary Angela O’Neal, David Yeomans, Nanette Santoro, Nina Riggins, Lars Edvinsson

**Affiliations:** 1 Association of Migraine Disorders, P.O. Box 870, North Kingstown, RI 02852, USA; 2 Department of Biological Sciences, NC State University, Raleigh, NC 27695, USA; 3 Division of Clinical and Translational Research, Department of Anesthesiology, Washington University School of Medicine, St. Louis, MO 63110, USA; 4 Brigham and Women’s Hospital, 45 Francis St, Boston, MA 02115, USA; 5 Department of Anesthesia, Pain and Perioperative Medicine, Stanford University School of Medicine, 300 Pasteur Drive, Stanford, CA 94305, USA; 6 Department of Obstetrics and Gynecology, University of Colorado School of Medicine, Aurora, CO 80045, USA; nanette.santoro@cuanschutz.edu; 7 Brain Performance Center and Research Institute, San Diego, CA 92122, USA; 8 Division of Experimental Vascular Research, Department of Clinical Sciences, Lund University Hospital, 22185 Lund, Sweden; lars.edvinsson@med.lu.se

**Keywords:** sex hormones, migraine, estrogen, oxytocin, progesterone, testosterone, prolactin, vasopressin

## Abstract

Sex hormones and migraine are closely interlinked. Women report higher levels of migraine symptoms during periods of sex hormone fluctuation, particularly during puberty, pregnancy, and perimenopause. Ovarian steroids, such as estrogen and progesterone, exert complex effects on the peripheral and central nervous systems, including pain, a variety of special sensory and autonomic functions, and affective processing. A panel of basic scientists, when challenged to explain what was known about how sex hormones affect the nervous system, focused on two hormones: estrogen and oxytocin. Notably, other hormones, such as progesterone, testosterone, and vasopressin, are less well studied but are also highlighted in this review. When discussing what new therapeutic agent might be an alternative to hormone therapy and menopause replacement therapy for migraine treatment, the panel pointed to oxytocin delivered as a nasal spray. Overall, the conclusion was that progress in the preclinical study of hormones on the nervous system has been challenging and slow, that there remain substantial gaps in our understanding of the complex roles sex hormones play in migraine, and that opportunities remain for improved or novel therapeutic agents. Manipulation of sex hormones, perhaps through biochemical modifications where its positive effects are selected for and side effects are minimized, remains a theoretical goal, one that might have an impact on migraine disease and other symptoms of menopause. This review is a call to action for increased interest and funding for preclinical research on sex hormones, their metabolites, and their receptors. Interdisciplinary research, perhaps facilitated by a collaborative communication network or panel, is a possible strategy to achieve this goal.

## 1. Introduction

Migraine is a neurological disorder affecting 12% of adults around the world at any one point in time [1]. Migraine symptoms can be different in women than men. Women can have more frequent and intense headaches with a higher risk of chronification [2]. Migraine is now recognized as the number one cause of disability globally for women aged 15–49 [3]. This gender difference in the behavior of migraine as a disease highlights the role of sex hormones in its pathophysiology. This review was the result of a round-table discussion among a panel of basic scientists from different disciplines on the topic of how sex hormones exert their effect on the nervous system, particularly migraine disease. The panelists were asked to discuss what the gaps in our knowledge were, what the barriers were, whether they could identify any new therapeutic agents that would provide an alternative treatment for migraine, and if there was an explanation for the clinical observation that the prevalence of some migraine-related symptoms, such as vestibular migraine and sinus pain and pressure, increase during perimenopause while headaches tend to recede.

## 2. Sex Hormone Fluctuation as a Trigger of Migraine

Migraine tends to follow a classic temporal pattern throughout a cisgender woman’s life that corresponds with sex hormone fluctuations during reproductive milestones in the female lifespan. Puberty is a key period with significant changes in sex hormone levels. Interestingly, in children and adolescents, the prevalence of migraine headaches is nearly equivalent in boys and girls [4], but during puberty, the prevalence of migraine between men and women diverges and is 3–4 times higher in women compared to men [5,6]. This sex difference corresponds to the onset of menarche and falls after menopause.

Migraine symptoms can be linked to menstrual cycle changes (menstrual migraine) and 18–25% of women with migraine experience migraine or headaches during menstruation [7]. Menstrual migraine can be associated with a higher frequency of migraine-accompanying symptoms and more frequent and severe migraine attacks [8]. A comparison of women with and without migraine shows that those with migraine are characterized by faster late-luteal-phase estrogen decline compared to women without migraine. Thus, the timing and rate of estrogen withdrawal has been proposed to be a marker of vulnerability to migraine in women [9]. Contraceptive pills reduce the number of migraine attacks, migraine days, pain scores, disability scores, and migraine medication use while reducing the frequency of aura, and lowering, but not eliminating, the risks of cardiovascular complications or other side effects [10,11,12]. Another strategy is to use estrogen supplementation with a pill, vaginal gel or patch during the menstrual week.

Migraine is a heterogeneous disease associated with many possible combinations of genetic defects which share a common phenotype of intermittent pain or other hypersensitivities. This accounts for the unpredictable response of migraineurs to medications and the effect of hormones on the nociceptive system is no exception. For some, a drop in estrogen triggers a menstrual migraine attack without aura; for others, high levels of estrogen can trigger an attack with aura [13].

Migraine disease has a complex relationship with pregnancy. For 8% of women with migraine, their headaches worsen during the first trimester. This is especially true for migraine without aura, which is more hormonally driven [14,15,16]. The majority of women with migraine generally experience reduced migraine symptoms by the third trimester [17]. However, many women have the acute onset of headaches during pregnancy. Approximately 60% of these new headaches will be related to migraine but caution must be taken to evaluate pregnant women for secondary headaches [18]. A third of women will have postpartum headaches [19]. For those who continue to have migraine symptoms during their pregnancy and immediately postpartum, treatment options are limited to protect the fetus. There are specific recommendations for safe care of women with migraine headaches during pregnancy and breastfeeding [20].

Perimenopause, the period of two to eight years when menses first become irregular prior to the year after the end of menses, is a time when hormonal fluctuations are still occurring, and pre-existing migraine symptoms can remain unchanged, improve, or worsen [21,22,23]. In total, 8–13% of women report their first migraine during perimenopause [24,25]. However, many women see a decrease in headache prevalence during this period [26,27], most prominently in women who already suffer from migraine with aura [28]. For unexplained reasons, mid-facial pain and pressure and vestibular migraine can become prominent symptoms during perimenopause and menopause [29]. Hormone replacement therapy, or menopause replacement therapy (MRT), usually a continuous dosing of estrogen alone or estrogen plus progestin (ethinyl estradiol 5 µg combined with norethindrone acetate 1 mg, estradiol 1 mg combined with 0.5 mg norethindrone acetate, or transdermal estradiol combined with one-quarter or one-half of a 5 mg norethindrone daily) [30], remains an option, particularly for those women who have not had a hysterectomy because estrogen alone increases the risk of endometrial cancer. Transdermal estrogen patches or gels can be efficacious and less risky than systemic estrogen replacement in treating migraine [7,23,31]. A significant shortcoming of supplemental hormone therapies is that they do not provide migraine relief for all women and, for some, headaches become more severe. But a second major shortcoming of MRT is that, although the dosing of sex hormones is roughly half that of birth control pills, the risks of heart disease, stroke, blood clots, and breast cancer are not eliminated [13,30,32].

The bottom line is that current sex hormone supplements play a valuable role in mitigating the symptoms of migraine, but, because they are still associated with serious complications, especially migraine with aura, and exacerbate migraine symptoms in some, many medical professionals choose not to use hormone supplements in their migraine treatment plan. For example, plant-derived hormones (phytoestrogens) and the derivative bio-identical hormones are effective in reducing menstrual-related migraine headaches [33], but there is no rigorous scientific evidence that these supplements are safer or more natural compared to the current hormonal interventions. Phytoestrogen-containing foods, such as soy, are recommended over supplements, and all phytoestrogens should be avoided if there is a chance of pregnancy because these compounds might adversely affect the endocrine system. It is speculated that they might be safer in older women, such as those suffering from menopausal symptoms, particularly hot flashes [34,35], but currently there is not enough evidence to conclude that the benefits of phytoestrogens outweigh their potential health risks [36], and they do not appear to be ideal migraine preventive agents. Thus, since many women with migraine are unable to find an effective preventive therapy, there remains the challenge to understand how sex hormone supplements work, with the goal that select metabolites or synthetic derivatives might be both efficacious and safer than current hormonal therapies.

## 3. Which Sex Hormones Should Be the Target?

### 3.1. Estrogen

Estrogen plays a complicated role in migraine disease. Both drops and fluctuations in estrogen are associated with migraine symptoms, but its effect varies between individuals because of different receptors, metabolites, and interactions with other hormones. The dominant understanding of how crucial estrogen is in protecting individuals from migraine symptoms is what happens when estrogen levels decline: the estrogen withdrawal hypothesis. This hypothesis theorizes that drops in plasma estrogen trigger migraine attacks and neuroinflammation, eventually leading to chronic sensitization [37]. There are several possible mechanisms to explain his theory. One explanation is that estrogen suppresses pain by binding to estrogen receptor alpha (ER alpha) and estrogen receptor beta (ER beta), which are primarily associated with cell nuclei in the trigeminal ganglia. Activation of these nuclear receptors regulates inflammatory genes that ultimately suppresses cell excitability [38]. Also, this hypothesis may be explained by drops in estrogen leading to higher levels of calcitonin gene-related peptide (CGRP) [23].

CGRP is believed to be among the critical neuropeptides responsible for the throbbing pain associated with a migraine attack and the neuroinflammation that causes both pain and that perhaps cause neuroplastic neural changes responsible for chronic central sensitization [39]. Specifically, estrogen may also increase neurogenic vasodilation and gene regulation. For example, in mice, expression of neuropeptide Y and galanin, two neuropeptides which may inhibit or modulate CGRP mechanisms in trigeminal neurons, may play a part in the fluctuations of head pain during the estrus cycle [40].

While the estrogen withdrawal hypothesis focuses primarily on the trigeminal nerves, it is important to recognize the wider-ranging actions of estrogen in other parts of the body and brain [41]. A second mechanism to explain the estrogen withdrawal theory was demonstrated in an animal model where reduced levels of estrogen were shown to increase the frequency of cortical spreading depressions, the electrophysiological event believed to be responsible for triggering the trigeminal system and headaches, as well as auras [42].

There are various mechanisms that might explain how cortical spreading depressions are initiated. For example, estrogen is known to rapidly alter cellular excitability and gene expression in hypothalamic neurons [43,44]. And estrogen affects energy homeostasis via the proopiomelanocortin (POMC) neurons in the hypothalamic arcuate [45], and may play a role in migraine. Other brain regions, such as the mesolimbic cortical reward system, have also been implicated and show profound estrogen sensitivity [46,47,48]. The complexity stems from having three forms of estrogen (estrone, estradiol and estriol), thirteen estradiol metabolites, and two classes of receptors with different isomers which are functionally distinct and differentially distributed throughout the brain. Estrogen has other metabolic functions that might contribute to pain control indirectly, such as its indirect effect on serotonin [49].

### 3.2. Progesterone

Progesterone, the second major sex hormone, is produced in the ovaries, adrenal glands and placenta, and primarily helps maintain pregnancy. Progesterone with estradiol is found at the onset of menstrual migraines. Nonetheless, it is more likely that the withdrawal of estradiol, rather than progesterone, initiates migraine headaches. Instead, progesterone appears to protect neurons by suppressing neuroinflammation and reducing trigeminal nerve sensitivity. In one study, the receptive field size of facial trigeminal mechanoreceptors was not increased by treatment with progesterone, unlike the effects of estradiol [50].

It may be in the interplay with additional factors where progesterone plays an integral role in pain modulation. In a longitudinal study of fibromyalgia, it was high levels of progesterone and testosterone together that were associated with less pain [51]. Progesterone and testosterone are able to penetrate the blood-brain barrier and function as precursors for neurosteroids. There is an example of a progesterone derivative which enhances GABA function by modulating GABA receptors and, in turn, inhibits neuronal sensitivity [52,53]. Furthermore, both progesterone and allopregnanolone appear to dampen nociception in the trigeminovascular system and to reduce neurogenic inflammation in migraine through neuron-glia interactions [52]. In addition, in animals, progesterone and estradiol affect two CNS pathways that lead to increased neuroprotection [54]. But the role of progesterone in neuroinflammation is complicated by the finding that, during menstruation, prostaglandins rise and promote neuroinflammation through the release of substance P, neurokinins, and CGRP [55].

Currently, synthetic progesterone is used as a form of birth control and a migraine preventive agent in the form of a continuous low dose of progestin. Bio-identical progesterone can be delivered in three formulations: orally, topically, and as a suppository. Progesterone may improve insomnia as a mild sedative, and improve sleep apneas by stimulating respiration [56]. Finally, the progesterone metabolite, allopregnanolone, plays a role in the disproportionate level of mood disorders in susceptible women [57], and may begin to explain the high prevalence of anxiety in those with migraine.

### 3.3. Testosterone

A popular belief is that testosterone is the male hormone whereas estrogen is the female hormone. However, this is an oversimplification, as both estrogen and testosterone have important roles to play in individuals of either sex [58]. In both males and females, the balance between estrogen and testosterone production throughout life influences the function of both reproductive and nonreproductive organs [58].

Testosterone could be a potential therapeutic target, as it has an antinociceptive effect [59,60,61,62,63]. In animal studies, after gonadectomy or the blocking of testosterone receptors, animals appeared more sensitive to nociceptive stimuli [64,65,66,67,68]. The few human studies performed support an analgesic effect of testosterone, as higher testosterone levels are associated with lower experimental pain sensitivity [69]. Studies on the relationship of testosterone to migraine are few. Testosterone levels are lower in adults with migraine vs. without migraine, and are related to migraine severity. Interestingly, even when similar testosterone levels are found, men with migraine more frequently report symptoms of androgen deficiency compared to men with no migraine. However, one study found that no differences in testosterone levels were found in women with vs. without migraine, and that migraine pain intensity was not correlated with testosterone levels. In addition, transgender subjects who were given androgen-blocking medication and estrogen replacement developed increased levels of migraine with aura, similar to the effect of estrogen replacement therapy in cisgender women [13]. Since men with lower levels of androgen are prone to cluster headaches [70], the androgen deficiency model of migraine is based on the premise that testosterone offers neuroprotection. This theory is complicated by finding that, in contrast to estrogen which promotes neuroinflammation through CGRP and other neuropeptides, testosterone promotes neuroinflammation through microglial pathways. Therefore, while testosterone supplementation in females might protect against progression to chronic migraine, it will not have the same effect due to the gender-specific physiology of males [71].

Testosterone appears to be able to effectively reduce symptoms by suppressing spreading depressions, increasing serotonin, stabilizing cerebral blood flow, and reducing cell excitability and neuroinflammation [72]. These metabolic effects may explain the findings that testosterone treatment can improve clinical pain and experimental pain sensitivity in patients with chronic pain, including in patients with temporomandibular joint pain, fibromyalgia, and migraine [73,74,75,76], and that testosterone treatment delivered by a subcutaneous implant significantly reduces migraine intensity [75]. Thus, although testosterone is not thought to play a causal role in migraine, it likely modulates pain. Nonetheless, limited evidence and complex effects are reasons that testosterone is not included in migraine management guidelines.

### 3.4. Oxytocin

Oxytocin’s (OT) therapeutic effects in migraine are complex and widespread in the nervous system, including at the level of the primary sensory neuron, spinal cord, and in a variety of brain regions associated with pain processing and modulation [77,78,79]. A recent theory is that menstrual migraines are related to a drop in both estrogen and OT during menstruation. Whether the lower concentrations of OT are secondary to the effect of less available estrogen in the CNS is not yet known.

The effect of OT on migraine has been shown via a case report in which intravenous OT provided analgesia and migraine relief [80]. In addition, double-blind, placebo-controlled clinical studies have shown evidence that intranasal OT sprays are efficacious for treating migraine pain in adult men and women [77,81] and experimental-evoked pain in men [82]. A benefit of oxytocin as a treatment for migraine is that it is routinely administered intranasally for inducing labor, postpartum care, and for enhancing lactation, and its safety profile is well documented. In addition, intranasal oxytocin in humans has no major side effects [83].

OT is a neuropeptide that exerts its pain-inhibitory effects both at the level of the primary afferent fiber and in the central nervous system. The first mechanism is via the descending neural pathway from the paraventricular nucleus (PVN) to the dorsal horn of the spinal cord [84,85]. Signals from the PVN release oxytocin in the spinal dorsal horn that activate GABAergic interneurons in the dorsal horn which secondarily recruit other inhibitory GABAergic interneurons and suppress pain signals carried by ascending A-delta and C-fibers [86,87,88,89]. The second mechanism is where OT released from the supraoptic nucleus (SON) in the hypothalamus, periaqueductal gray (PAG), rostral ventromedial medulla (RVM), and the spinal dorsal horn [90,91] modulates central endogenous pain pathways by raising nociceptive thresholds [92,93]. OT can suppress headache pain by binding to oxytocin receptors (OTRs) specifically in the trigeminal nucleus and trigeminal ganglia [94]. Imaging studies of migraine patients show overlap in the localization of OT/OTR, particularly those in the brainstem, thought to be migraine generators [95].

OTR mRNA and proteins are expressed in nociceptive C-fibers and Aδ-fibers in the adult rat trigeminal ganglia [94], and have a high level of co-expression with CGRP in trigeminal ganglia neurons [77]. OT dose-dependently blocks the release of calcitonin gene-related peptide (CGRP) from trigeminal afferent neurons innervating the dura in vitro [94]. CGRP is critical for the pathogenesis for chronic migraine, meaning that OTR activation on trigeminal nociceptive neurons could be a key mechanism of decreased headache intensity and frequency in migraine. 

OT might have a general anti-inflammatory effect in orofacial nociceptive pathways by activating OTR, which can also suppress pro-inflammatory markers IL-1B and TNFa in the trigeminal ganglia (and in the spinal trigeminal nucleus caudalis) by inhibiting upregulation of these cytokines. A secondary effect is that inflammatory pain stimulates increased OTR gene expression [96]. But with less OT, trigeminal ganglia neurons become more sensitive, enhancing the likelihood of a migraine being triggered [97].

### 3.5. Vasopressin

Arginine vasopressin (AVP) is a neuropeptide hormone that has an antidiuretic effect in low concentrations, but at higher concentrations it causes vasoconstriction. Together, these effects raise blood pressure. AVP also has a role in pain, behavior, platelet aggregation, and blood coagulation functions. Specifically, AVP, in response to stress and pain, may be relevant to migraine pathophysiology [98,99]. Platelets have more AVP receptors in women who experience migraine [100]. It is possible that the AVP secretion has nothing directly to do with migraine, but, since the highest levels of AVP during a migraine attack may be associated with emesis [101] and vomiting, hypovolemia and nausea without vomiting trigger AVP release. Elevated levels of AVP may be responsible for the facial pallor, antidiuresis, and coagulation abnormalities occasionally observed in migraine [102]. In addition, some migraine precipitators (stress, ethanol, etc.) cause decreased AVP secretion and bioavailability, while some migraine-improving factors (tricyclic antidepressants, sleep, etc.) are associated with an increase in AVP [103]. Intranasal delivery of AVP has been described as an effective therapeutic agent for headache control [104].

Much of AVP is synthesized in the SON of the hypothalamus and, while AVP is largely stored in and secreted from the pituitary, AVP-containing hypothalamic fibers are widely distributed in the CNS [105]. These fibers reach different centers in the brainstem and, in particular, the trigemminal nuclei. The AVP receptors (VP1 and VP2) are found in the trigeminal ganglion [94]. Thus, the AVP system has many ways to modulate migraine pathophysiology. Since there are no direct fibers containing AVP in the trigeminovascular system, it is likely that the peptide may diffuse into this system. Overall, there exists sufficient evidence to maintain interest in the use of AVP to moderate the onset of headaches [106]. 

### 3.6. Prolactin

Prolactin (PRL) is a hormone that is responsible for lactation, breast development, and hundreds of other actions needed to maintain homeostasis. PRL is chemically related to growth hormones and placental lactogen hormones. In an animal model, high levels of prolactin increased meningeal trigeminal pain sensitivity by only affecting CGRP in female rodents [107]. In humans, serum prolactin levels are higher in those with migraine. Individuals with prolactin-secreting pituitary adenomas were found to have a higher incidence of headaches and migraine attacks [108]. With monoclonal antibodies targeting prolactin receptors, a recent report opens new possibilities to better understand the complex interaction between prolactin and CGRP, but blocking prolactin receptors in humans poses risks of interfering with the other functions of this hormone [109].

## 4. Limitations of Current Methods

Advances in understanding sex hormones in humans are hampered by the challenges of reliably creating an equivalent model of a migraine attack and measuring responses to interventions in animal models. Additionally, the translational value of preclinical studies can be uncertain due to a predominant use of males or not reporting sex as a biological variable [110,111], a reliance on ovariectomies, and modeling hormonal changes in animals that have an estrous cycle rather than a human-like menstrual cycle [112]. Furthermore, the effect of sex hormones on migraine and pain may vary depending on the pain model, model species, and experimental design in laboratory settings. The expert panel identified the lack of an established migraine animal model as one of the barriers to rapid progress in migraine research. For human research, the design of effective human studies has been challenging. Blood sampling of hormone levels is complicated by fluctuation throughout the day and month. The differential effect of sex hormone interventions might be impacted by the delivery method, timing of delivery, and dose, as well as sex, age and other conditions and medications of the patients.

As migraine is inherently a complex disorder involving different biological systems including the nervous, endocrine, endothelial, and immune systems, an interdisciplinary and collaborative approach among clinical and preclinical researchers is encouraged. Furthermore, given the limited number of basic scientists exploring this subject, it is critical that there is a cross-pollination of knowledge and ideas for research between often isolated fields of study. For example, chronic pain, which includes fibromyalgia, back pain, and TMJ overlaps with research performed in immunology, headache medicine, and other medical specialties [113,114]. It will take a dramatic increase and maintained effort in advocacy and support from patients and medical professionals to advance our knowledge of migraine and hormonal pathophysiology enough to lead to hormonal therapies of greater precision and safety.

## 5. Conclusions and Future Directions

While a large body of research has established hormonal changes and fluctuations as a driver of migraine symptoms in women and transgender people, the relation to hormonal life events is not definitively known for the full range of migraine symptoms. A new HEADS (headache, ear, auditory, dizziness, and sinus) Registry is now available to record and track many of these symptoms (reference: headsregistry.lumiio.com). Additionally, clarification of the mechanisms behind the emergence and recession of different migraine-related symptoms remains. Hormones may have both a causal role in migraine generation and also contribute to pain propagation.

This panel identified several potential hormones and mechanisms that show promise for improved migraine therapeutics, but the conclusion was that more resources need to be concentrated on this significantly debilitating neurovascular condition. In particular, the gender-specific nature of migraine disease calls for the need to better understand how hormones affect the nervous system. New areas of research are required to better understand the mechanisms by which sex hormones relate to changes in migraine symptoms during the periods of hormonal fluctuation in puberty, menstruation, pregnancy, and perimenopause. Translationally relevant animal models of migraine will play a key role in providing mechanistic insights, especially when coupled with clinical data. We highlight the theoretical opportunity to create novel hormone-based therapeutic molecules that might desensitize the hyperactive migraine nervous system without the potential side effects of contraceptives and hormone replacement therapy. Moreover, progress in understanding how hormones affect the nervous system will lead to innovations in treating not only migraine, but other menopausal symptoms.

## Data Availability

No new data were created or analyzed in this study. Data sharing is not applicable to this article.
